# Changes in Emergency Department Activity and the First COVID-19 Lockdown: A Cross-sectional Study

**DOI:** 10.5811/westjem.2021.2.49614

**Published:** 2021-05-07

**Authors:** Kate Honeyford, Charles Coughlan, Ruud G. Nijman, Paul Expert, Gabriel Burcea, Ian Maconochie, Anne Kinderlerer, Graham S. Cooke, Ceire E. Costelloe

**Affiliations:** * Imperial College London, Department of Primary Care and Public Health, Global Digital Health Unit, London, United Kingdom; † Imperial College Healthcare NHS Trust, Department of Paediatrics, London, United Kingdom; ‡ St. Mary’s Hospital, Imperial College Healthcare NHS Trust, Department of Paediatric Emergency Medicine, London, United Kingdom; § Imperial College London, Section of Paediatric Infectious Diseases, London, United Kingdom; ¶ St. Mary’s Hospital, Imperial College Healthcare NHS Trust, London, United Kingdom; || Imperial College London, Department of Infectious Disease, London, United Kingdom; # Imperial Biomedical Research Centre, London, United Kingdom

## Abstract

**Introduction:**

Emergency department (ED) attendances fell across the UK after the ‘lockdown’ introduced on 23rd March 2020 to limit the spread of coronavirus disease 2019 (COVID-19). We hypothesised that reductions would vary by patient age and disease type. We examined pre- and in-lockdown ED attendances for two COVID-19 unrelated diagnoses: one likely to be affected by lockdown measures (gastroenteritis), and one likely to be unaffected (appendicitis).

**Methods:**

We conducted a retrospective cross-sectional study across two EDs in one London hospital Trust. We compared all adult and paediatric ED attendances, before (January 2020) and during lockdown (March/April 2020). Key patient demographics, method of arrival, and discharge location were compared. We used Systemised Nomenclature of Medicine codes to define attendances for gastroenteritis and appendicitis.

**Results:**

ED attendances fell from 1129 per day before lockdown to 584 in lockdown, 51.7% of pre-lockdown rates. In-lockdown attendances were lowest for under-18s (16.0% of pre-lockdown). The proportion of patients admitted to hospital increased from 17.3% to 24.0%, and the proportion admitted to intensive care increased fourfold. Attendances for gastroenteritis fell from 511 to 103, 20.2% of pre-lockdown rates. Attendances for appendicitis also decreased, from 144 to 41, 28.5% of pre-lockdown rates.

**Conclusion:**

ED attendances fell substantially following lockdown implementation. The biggest reduction was for under-18s. We observed reductions in attendances for gastroenteritis and appendicitis. This may reflect lower rates of infectious disease transmission, although the fall in appendicitis-related attendances suggests that behavioural factors were also important. Larger studies are urgently needed to understand changing patterns of ED use and access to emergency care during the coronavirus 2019 pandemic.

## INTRODUCTION

The emergence of COVID-19 and subsequent ‘lockdown’ introduced by the British Government on 23^rd^ March 2020^1^ had a substantial impact on emergency department (ED) attendances. Total ED attendances in England in March 2020 fell by 29.4% year-on-year.^2^ The reasons for this change in ED activity are likely to be multifactorial. To reduce pressure on EDs, patients were instructed to seek advice from online resources and National Health Service (NHS) telephone services. The closure of schools and workplaces is likely to have led to a reduction in the spread of infectious diseases.^2^ Reductions in organised sports and recreational activity have previously been linked to reductions in physical injuries.^3^ It is suggested that reductions in children attending EDs reflect parents’ concerns about acquiring nosocomial COVID-19.^4^ Several authors have highlighted the potential for collateral damage from lockdowns, with patients deterred from seeking help for serious injuries and illnesses at risk of poorer outcomes.^5^,^6^

**Figure 1:**
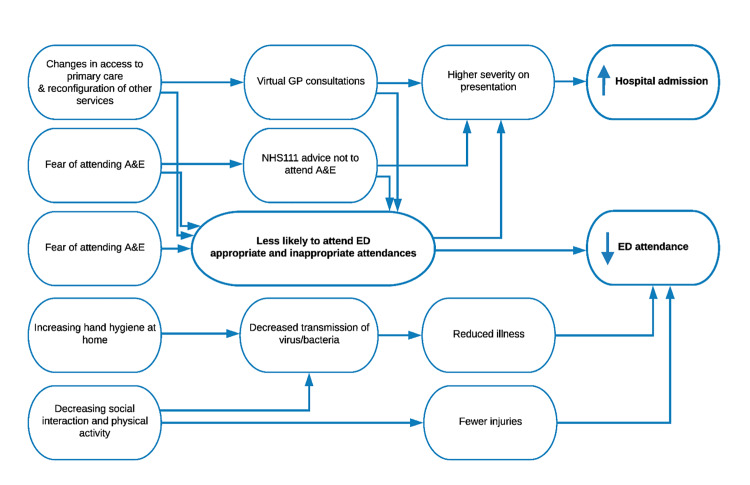
Conceptual framework for changes in emergency department attendance associated with ‘lockdown’ for conditions unrelated to COVID-19. *ED*, emergency department.

We hypothesised that the impact of the first nationwide lockdown on ED attendance would vary by patient demographics and clinical reason for attendance. We have proposed causal pathways leading to changes in ED attendances and hospital admissions. These are summarised in Figure 1. Using a snapshot of ED data, we examined the number of pre- and in-lockdown ED attendances for two COVID-19 unrelated diagnoses:

- Gastroenteritis – an infectious disease which we would expect to be affected by lockdown measures.
- Appendicitis – an acute disorder which we would expect to be largely unaffected by lockdown measures.

## METHODS

Emergency department attendance information was provided by the NHS Trust data warehouse team. Information included patient information: age, gender, ethnicity, residential partial postcode, arrival mode at ED, destination at discharge and Systemised Nomenclature of Medicine Clinical Terms (SNOMED-CT)^7^ diagnostic codes. At the hospital Trust sites, diagnostic codes are entered by the treating ED clinician immediately after conducting a clinical assessment. The code is based on their clinical impression from history and examination, blood tests and, where applicable, specialist imaging investigations. Data was extracted from the ED part of the electronic health system (Cerner Corporation) by the Trust data management team and transferred as a table into a secure analysis environment.

We selected a four-week period (6^th^ January 2020–2^nd^ February 2020) as the pre COVID-19 phase and a four-week period (23^nd^ March 2020–19^th^ April 2020) as the in-lockdown period. We included all ED attendances (children and adults). We compared ED attendances based on patient demographics, and we compared attendances for gastroenteritis and appendicitis, using the SNOMED-CT codes in Appendix 1 to define the study population, in total and by age, to assess examples of one diagnosis likely to be affected and one diagnosis unlikely to be affected by lockdown measures. Differences were assessed using chi-squared test. Analysis was done in R version 3.60 (The R Project for Statistical Computing, Vienna, Austria). This study was granted service evaluation approval through Imperial College London NHS Trust (Ref:228). Patients or the public were not involved in the design, or conduct, or reporting, or dissemination plans of our research.

## RESULTS

There were 31,624 ED attendances in the pre-lockdown period and 16,355 in-lockdown, a reduction from 1129 attendances a day pre-lockdown, to 584 a day in-lockdown. Arrivals in ambulances accounted for 61.2% attendances pre- and 51.7% in-lockdown. As a proportion of pre-lockdown attendances, in-lockdown attendances were lowest for under-18s (16.0%) and highest for patients aged 40–60 (76.7%). Male and Asian patients made up a higher proportion of in-lockdown than pre-lockdown attendances. This was also true for patients from postcodes considered the primary catchment for the Trust (77% pre-lockdown and 80% in-lockdown). Pre-lockdown, 17.5% of ED attendances resulted in admission to inpatient wards or intensive care units (ICU), compared to 24.4% in-lockdown. Following lockdown implementation, 4% of admitted patients were admitted directly to ICU, compared to 1% pre-lockdown. Changes in attendances were deemed statistically significant (*P*<0.0001, chi-squared test). Results are summarised in Table 1.

### Gastroenteritis and Appendicitis Attendances Pre- and In-lockdown

Pre-lockdown, there were 511 attendances with a gastroenteritis code, 1.62% of all attendances, compared to 103 attendances in-lockdown, 0.61% of the total. Total ED attendances with an appendicitis code also decreased over the study period, from 144 (0.46% total) to 41 (0.24% total). Attendances for gastroenteritis in-lockdown were 20.2% of pre-lockdown, compared to 28.5% for appendicitis. While a similar proportion of patients with gastroenteritis were directly discharged home in both time periods (84% before lockdown compared to 83% in lockdown), we observed a threefold increase in discharge rates among patients with appendicitis following lockdown implementation (13% compared to 34%).

Changes in attendances for both diseases varied with age. We observed the most significant reduction in attendances with gastroenteritis amongst children and young people and patients aged over 60.

## DISCUSSION

In line with national data,^2^ we found that overall ED attendances almost halved since the introduction of lockdown. Similar to other reports,^6^,^8^ the impact of the lockdown on ED attendance rate was greatest in the under-18’s, suggesting changes in parental health-seeking behaviour. Following lockdown implementation, a higher proportion of ED patients required hospital admission, and there was a fourfold increase in the proportion admitted directly to ICU. These changes may reflect patients attending with more serious conditions, severe COVID-19 and/or the increase in ICU capacity. In contrast, for patients with appendicitis there was a reduction in admissions, which was likely due to a change in clinical management to antibiotics during this time.^9^

We hypothesised a reduction in gastroenteritis-related attendances following lockdown implementation due to reduced interpersonal contact and spread of infectious diseases. The results show that attendances in-lockdown fell to one-fifth of pre-lockdown rates. A reduction in ED attendances for specific infectious diseases has been described in England^2^ and Italy.^10^ However, the decrease in attendances for appendicitis suggests that reduced transmission alone cannot explain the reduction in ED attendances seen after lockdown.

## LIMITATIONS

Not all of the differences we have reported are attributable to lockdown. Seasonal variations are seen in a range of infectious diseases, including gastroenteritis. However, seasonal variations alone are unlikely to account for our findings as nationally published data for this hospital Trust suggest April attendances in 2019 were only 5% lower than January 2019.^11^ Gastrointestinal (GI) symptoms are seen in around 15% of children and adults with confirmed SARS-CoV-2 infection.^12^ As such, patients with COVID-19 presenting with fever and prominent GI symptoms may be wrongly diagnosed with gastroenteritis, confounding our in-lockdown results. We did not exclude these presentations as we did not know patients’ motivations for attending ED, and testing policy evolved over the study period. Nonetheless, we feel this confounding effect is unlikely to have had a substantial bearing on our results as we observed a fourfold reduction in gastroenteritis attendances during the in-lockdown period. Indeed, if large numbers of patients with GI symptoms secondary to COVID-19 were wrongly labelled as having infectious gastroenteritis, we may have underestimated the extent of the fall in ED attendances for gastroenteritis across the study period.

Treatment pathways have changed during the pandemic as hospitals have sought to minimize non-emergency surgery. This includes an increase in the management of uncomplicated appendicitis with oral antibiotics possibly explaining the fall in appendicitis-related admissions.^8^ However, this cannot explain the reduction in ED attendances with appendicitis to less than one-third of pre-lockdown levels, as there was no concurrent change in advice to primary care practitioners and patients regarding appendicitis during this time.

## CONCLUSION

We have developed a conceptual causal framework proposing various factors which may lead to lower ED attendances during the COVID-19 pandemic. These factors include deterred care seeking due to fears of acquiring infection in hospital settings and patients seeking health advice from other services. Future studies, using larger, more generalisable data from across whole healthcare systems must aim to untangle the relative contributions of these different factors and ensure that sick patients have timely and equitable access to emergency care.

## Supplementary Information



## Figures and Tables

**Table 1:** Characteristics of patients attending two emergency departments in one hospital Trust in North West London.

Time period	Pre-lockdown	In-lockdown	

	n (% of all attendances)	n (% of all attendances)	as % of pre-lockdown attendance
Total number of attendances	31,624	16,355	51.7%
Male	15,359 (48.6%)	8,870 (52.9%)	57.8%
Age			
0–18	7,054 (22.2%)	1,131 (6.8%)	16.0%
18–40	8,412 (26.6%)	4,865 (29.0%)	57.8%
40–50	3,073 (9.7%)	2,368 (14.1%)	77.1%
50–60	3,616 (11.4%)	2,702 (16.1%)	74.7%
60–70	2,922 (9.2%)	1,868 (11.1%)	63.9%
70–85	4,563 (14.4%)	2,797 (16.7%)	61.3%
85+	1,984 (6.3%)	1,034 (6.2%)	52.1%
Ethnic group			
Any other ethnic group	7,704 (24.4%)	3,787 (22.6%)	49.2%
White	12,576 (39.8%)	6,342 (38.8%)	50.4%
Black or Black British	4,256 (13.5%)	2,239 (13.7%)	52.6%
Asian	1,499 (4.7%)	1,208 (7.2%)	80.6%
Not stated	3,445 (10.9%)	2,154 (12.9%)	62.5%
Not known	547 (1.7%)	594 (3.5%)	108.6%
Catchment area	24,568 (77.7%)	13,467 (80.3%)	54.8%
Arrival by ambulance	19,340 (61.2%)	7,972 (51.70%)	41.2%
Discharge destination			
Home^1^	20,935 (66.2%)	10,930 (66.8%)	52.2%
Admitted to hospital	5,463 (17.3%)	3,921 (24.0%)	71.8%
Other hospital care^2^	3,945 (12.5%)	798 (4.9%)	20.2%
Mortuary	38 (0.1%)	69 (0.4%)	181.6%
Missing	259 (0.8%)	619 (3.7%)	239.0%
Disease profile			
Gastroenteritis	511 (1.6%)	103 (0.6%)	20.2%
Appendicitis	144 (0.5%)	41 (0.3%)	28.5%

1 Home includes nursing and residential care homes.

2 Other hospital care includes ambulatory care centre and short stay wards.

P<0.0001 for all pre- vs in-lockdown comparisons.
