# Supplementary material for: Changes in Emergency Department Activity and the First COVID-19 Lockdown: A Cross-sectional Study
Source: West J Emerg Med. 2021 May 7;22(3):603–7. doi: 10.5811/westjem.2021.2.49614 (PMC8203011; doi:10.5811/westjem.2021.2.49614)
Supplement: Supplementary file 1 [file wjem-22-603-s001.docx]

Supplementary Materials – SNOMED-CT codes

**Table 1a.** Appendicitis.

| SNOMED-CT Code | SNOMED-CT Description |
| --- | --- |
| 62224002 | Abscess of appendix (disorder) |
| 85189001 | Acute appendicitis (disorder) |
| 266439004 | Acute appendicitis with appendix abscess (disorder) |
| 28845006 | Acute appendicitis with generalized peritonitis (disorder) |
| 698294004 | Acute appendicitis with localized peritonitis (disorder) |
| 51036000 | Acute appendicitis with peritoneal abscess (disorder) |
| 196781001 | Acute appendicitis with peritonitis (disorder) |
| 72048003 | Acute appendicitis without peritonitis (disorder) |
| 235769005 | Acute focal appendicitis (disorder) |
| 84534001 | Acute fulminating appendicitis (disorder) |
| 64994000 | Acute fulminating appendicitis with perforation AND peritonitis (disorder) |
| 50846009 | Acute gangrenous appendicitis (disorder) |
| 64252005 | Acute gangrenous appendicitis with perforation AND peritonitis (disorder) |
| 4998000 | Acute obstructive appendicitis (disorder) |
| 28358004 | Acute obstructive appendicitis with perforation AND peritonitis (disorder) |
| 286967008 | Acute perforated appendicitis (disorder) |
| 735591005 | Acute phlegmonous appendicitis (disorder) |
| 235770006 | Acute suppurative appendicitis (disorder) |
| 26826005 | Amebic appendicitis (disorder) |
| 49438003 | Appendectomy with drainage (procedure) |
| 74400008 | Appendicitis (disorder) |
| 91313006 | Appendicitis of a pelvic appendix (disorder) |
| 5596004 | Atypical appendicitis (disorder) |
| 58997001 | Chronic appendicitis (disorder) |
| 418171008 | Complicated appendicitis (disorder) |
| 174036004 | Emergency appendectomy (procedure) |
| 174039006 | Emergency excision of normal appendix (procedure) |
| 1299000 | Excision of appendiceal stump (procedure) |
| 80146002 | Excision of appendix (procedure) |
| 443935000 | Excision of ruptured appendix by open approach (procedure) |
| 123601005 | Focal appendicitis (disorder) |
| 82730006 | Incidental appendectomy (procedure) |
| 174045003 | Interval appendectomy (procedure) |
| 6025007 | Laparoscopic appendectomy (procedure) |
| 708876004 | Laparoscopic appendectomy using robotic assistance (procedure) |
| 174041007 | Laparoscopic emergency appendectomy (procedure) |
| 307581005 | Laparoscopic interval appendectomy (procedure) |
| 235313004 | Non-emergency appendectomy (procedure) |
| 67365005 | Recurrent appendicitis (disorder) |
| 6503008 | Relapsing appendicitis (disorder) |
| 25598004 | Retrocecal appendicitis (disorder) |
| 32084004 | Retroileal appendicitis (disorder) |
| 95547004 | Ruptured suppurative appendicitis (disorder) |
| 9124008 | Subacute appendicitis (disorder) |
| 42640003 | Suppurative appendicitis (disorder) |

**Table 1b.** Gastroenteritis.

| SNOMED-CT Code | SNOMED-CT Description |
| --- | --- |
| 69776003 | Acute gastroenteritis (disorder) |
| 359613008 | Acute infectious nonbacterial gastroenteritis (disorder) |
| 36789003 | Acute infective gastroenteritis (disorder) |
| 236063005 | Adenoviral gastroenteritis (disorder) |
| 707222009 | Epidemic gastroenteritis (disorder) |
| 416482004 | Food-borne gastroenteritis (disorder) |
| 25374005 | Gastroenteritis (disorder) |
| 713186008 | Gastroenteritis caused by drug (disorder) |
| 143000000000000 | Gastroenteritis caused by H1N1 influenza (disorder) |
| 10600000000000000 | Gastroenteritis caused by influenza (disorder) |
| 10600000000000000 | Gastroenteritis caused by Influenza A virus (disorder) |
| 57419008 | Gastroenteritis presumed infectious (disorder) |
| 240332005 | Infantile gastroenteritis (disorder) |
| 308119005 | Infantile viral gastroenteritis (disorder) |
| 12463005 | Infectious gastroenteritis (disorder) |
| 421454008 | Infectious gastroenteritis associated with acquired immunodeficiency syndrome (disorder) |
| 713570009 | Infectious gastroenteritis co-occurrent with human immunodeficiency virus infection (disorder) |
| 707352004 | Institution-acquired gastroenteritis (disorder) |
| 359651008 | Nonbacterial gastroenteritis of infant (disorder) |
| 12574004 | Noninfectious gastroenteritis (disorder) |
| 421983003 | Noninfectious gastroenteritis associated with acquired immunodeficiency syndrome (disorder) |
| 111843007 | Viral gastroenteritis (disorder) |
| 415822001 | Viral gastroenteritis caused by Rotavirus (disorder) |
